# Fast Hollow Fiber Liquid-Phase Microextraction as a Greener Alternative for the Determination of N,N-Dimethyltryptamine and Harmala Alkaloids in Human Urine

**DOI:** 10.3389/fchem.2020.558501

**Published:** 2020-10-07

**Authors:** Gabriela de Oliveira Silveira, Felipe Rebello Lourenço, Vitor Bruno, Mauricio Yonamine

**Affiliations:** ^1^Department of Clinical and Toxicological Analyses, School of Pharmaceutical Sciences, University of São Paulo, São Paulo, Brazil; ^2^Department of Pharmacy, School of Pharmaceutical Sciences, University of São Paulo, São Paulo, Brazil

**Keywords:** liquid-phase microextraction, green analytical chemistry, ayahuasca, DMT, harmala alkaloids, LC-MS/MS

## Abstract

Ayahuasca tea is an entheogen hallucinogenic beverage used for shamanic and spiritual purposes, prepared by the decoction of different Amazonian plants containing N,N-dimethyltryptamine (DMT) and harmala alkaloids. Since the therapeutic potential of this tea has been broadly studied in recent years, mainly for the treatment of psychiatric disorders, the determination of the ayahuasca tea components in human and animal matrices is of utmost importance. In order to avoid the use of large amounts of toxic solvents, typically employed in traditional sample preparation methods, hollow fiber liquid-phase microextraction (HF-LPME) presents a greener and time-saving alternative. The present study aims to fully develop and apply an HF-LPME method for the determination of DMT, harmine (HRM), harmaline (HRL), and tetrahydroharmine (THH) in human urine samples using liquid chromatography-tandem mass spectrometry (LC-MS/MS). Fractional factorial and Box–Behnken designs were used to identify and optimize significant method variables. Once optimized, validation has shown a limit of detection (LoD) of 1.0 ng/ml for DMT and 2.0 ng/ml for the harmala alkaloid. The limit of quantification (LoQ) was of 5.0 ng/ml for all analytes. The method has shown to be linear over a concentration range of 5–200 ng/ml (*r*^2^ ≥ 0.99). Intra/inter-day precision and accuracy met the acceptance criteria at the three quality control (QC) levels studied (15.0, 90.0, and 170.0 ng/ml, *n* = 6, each). Matrix effect evaluation showed predominant ion enhancement and recovery values were above 80%. Dilution factors of 10- and 20-fold have shown acceptable values of accuracy. Selectivity studies showed no interferences. Analysis of eight authentic samples collected from four subjects proved method feasibility. A simple, time-saving and green alternative for the analysis of DMT and harmala alkaloids in human urine samples was developed, optimized using design of experiments, fully validated and applied to authentic samples.

## Introduction

Ayahuasca tea is an entheogen preparation historically used by the indigenous people from the Amazon Basin region for at least 1,000 years (Callaway et al., 1999; Miller et al., 2019; Orsolini et al., 2020). In the last decades, the use of ayahuasca has spread itself across the world via neoshamanic groups, which has caught the attention of the scientific community regarding its possible risks and benefits (Riba et al., 2003; Oliveira et al., 2010a,b; de Oliveira et al., 2011; Sánchez and Bouso, 2015; Palhano-Fontes et al., 2017; Estrella-Parra et al., 2019). The beverage is prepared by the decoction of the *Banisteriopsis caapi* vine and, typically, the *Psychotria viridis* leaves; while the former is rich in the β-carboline harmala alkaloids (harmine, harmaline, and tetrahydroharmine), the latter is the N,N-dimethyltryptamine (DMT)-containing plant and the main responsible for its hallucinogenic effects (Callaway et al., 1999; McKenna, 2004; Tupper, 2008). As DMT is orally inactive, a synergistic effect takes place when the harmala alkaloids, also present in the brew, act as reversible MAO-A inhibitors in the gastrointestinal tract and block the degradation of DMT, which allows for the psychoactive substance to reach its serotoninergic receptors in the central nervous system (Riba et al., 2003; Tupper, 2008). In addition, it has been proven that tetrahydroharmine (THH) is a weak serotonin reuptake inhibitor (Callaway et al., 1999; McKenna, 2004; Frecska et al., 2016). Because of these unique features and the possible psychoplastogenic properties of DMT, several research groups have been studying the therapeutic potential of ayahuasca in the context of psychedelic therapy, mainly for the treatment of depression, anxiety, and drug dependence, as recently reviewed (Frecska et al., 2016; Muttoni et al., 2019; dos Santos and Hallak, 2020; Dunlap et al., 2020; Orsolini et al., 2020; Reiff et al., 2020).

As the relevance of using ayahuasca for both religious and scientific purposes expands continuously, the need for suitable analytical approaches rises accordingly. In fact, some methods for the analysis of ayahuasca alkaloids in plasma, whole blood, urine, hair, and sweat specimens have been published (Oliveira et al., 2012; de Morais et al., 2018; Simão et al., 2019; Tavares et al., 2020). The traditional liquid–liquid extraction (LLE) and solid-phase extraction (SPE) were formerly employed for the analysis of DMT or harmine (HRM) and harmaline (HRL) in urine specimens (Ishii et al., 1997; Forsström et al., 2001; Frison et al., 2008; Wang et al., 2009). However, the methods employed then used significantly large amounts of organic solvents, such as chloroform, ethyl acetate, hexane, and dichloromethane (Ishii et al., 1997; Forsström et al., 2001; Frison et al., 2008; Wang et al., 2009). The improvement in the sensitivity of chromatographic and mass spectrometry techniques has resulted in the need for less laborious sample preparation methods, such as dilute-and-shoot (Bjornstad et al., 2009; Mcilhenny et al., 2011; Pope et al., 2019) and protein precipitation (Zhao et al., 2012; Meyer et al., 2014; de Morais et al., 2018) approaches, which use very small amounts of urine. These simple, faster, cheaper, and less hazardous methodologies have become prominent in the analysis of ayahuasca alkaloids. Nonetheless, it is important to note that the reported protein precipitation methods still required the use of significant amounts of acetonitrile (Zhao et al., 2012; Meyer et al., 2014) and methyl *tert*-butyl ether (de Morais et al., 2018).

Consequently, in order to fulfill the requirements from the Green Analytical Chemistry (GAC), once the sample preparation step can hardly be obviated, novel developed methods must focus on the reduction, replacement, or elimination of the use of toxic organic solvents and reagents, optimization of energy consumption, and adequate waste management, which in return may improve the safety of the operator and provide cost-effective alternatives (Gałuszka et al., 2013; Spietelun et al., 2014; Armenta et al., 2015; Silveira et al., 2019). Sample preparation based on GAC proves to be an even more challenging step for it must guarantee sensitivity, recovery, accuracy, and reproducibility as well as ensuring minimal environmental impact (Gałuszka et al., 2013; Turner, 2013; Silveira et al., 2019). Among the several available strategies, the miniaturization of extraction systems is one of the most remarkable alternatives as it proves to be able to drastically reduce the volumes of both solvents and samples, from milliliters used on classic extraction procedures (such as LLE and SPE) down to microliters, or even to completely eliminate the use of such solvents, as in hollow fiber liquid-phase microextraction (HF-LPME) and solid-phase microextraction (SPME) (Spietelun et al., 2014; Silveira et al., 2019).

HF-LPME is a successful microextraction technique first introduced by Pedersen-Bjergaard and Rasmussen (1999). This technique is based on the use of porous polypropylene hollow fibers that are impregnated with 15–20 μl of an organic solvent within its pores to produce a supported liquid membrane (SLM) (Pedersen-Bjergaard and Rasmussen, 2005; Carasek and Merib, 2015). After that, the HF lumen is filled with an appropriate solution (acceptor phase), the SLM is introduced into the aqueous sample (donor phase), and the system is usually agitated in order to promote analyte transfer from the donor phase through the SLM into the acceptor phase, which is collected and submitted to further analysis (Pedersen-Bjergaard and Rasmussen, 2005; Silveira et al., 2019). If both the SLM and the acceptor solution are composed by the same organic solvent, the system is named two-phase LPME; on the contrary, the acceptor solution may be produced using an acidic or basic solution able to ionize the analytes—present in the donor phase mainly in its deionized form—which promotes analyte concentration within the fiber lumen by ion trapping (three-phase LPME) (Pedersen-Bjergaard and Rasmussen, 2005; Carasek and Merib, 2015).

The use of HF-LPME has been shown to offer a great performance and considering the importance of quantifying ayahuasca alkaloids in biological matrices, this study presents a fast HF-LPME method followed by ultra-high performance liquid chromatography-tandem mass spectrometry (UHPLC-MS/MS) for the determination of DMT, THH, HRL, and HRM in human urine samples. The current study has aimed to provide a simple miniaturized method in compliance with GAC requirements as an alternative to protein precipitation and dilute-and-shoot approaches which may not offer sufficient sensitivity, matrix clean-up, and sustainability. The HF-LPME method has been optimized through the use of design of experiments; it was then fully validated and its feasibility proof was achieved by the analysis of eight authentic samples donated by four individuals after a usual ayahuasca ceremony.

## Materials and Methods

### Reagents, Standards, and Materials

N,N-Dimethyltryptamine was purchased from Cerilliant Corporation (Round Rock, Texas, USA). HRM and HRL were acquired from Sigma-Aldrich (Saint Louis, USA). The internal standard, deuterated dimethyltryptamine (DMT-*d*_6_), was synthetized as described by Oliveira and collaborators (Oliveira et al., 2012). THH was synthetized from HRL, according to the method described by Callaway et al. (1996). DMT stock solution was available at 1.0 mg/ml while DMT-*d*_6_ [internal standard, (IS)], THH, HRL, and HRM powders were weighted out to produce methanolic stock solutions at that same concentration. When appropriate, standard solutions were simply diluted 10-, 100-, or 1,000-fold with methanol in volumetric glassware to obtain working solutions at concentrations of 100, 10.0, or 1.0 μg/ml. All standards solutions were stored in freezer at −20°C.

Methanol HPLC grade, n-octanol, n-non-anol, n-decanol, sodium bicarbonate, sodium carbonate, sodium borate, sodium chloride, ammonium formate, and formic acid (98–100% grade) were purchased from Merck KGaA (Darmstadt, Germany). A Milli-Q system was used to produce ultra-pure water (Millipore, Billerica, Massachusetts).

Hollow-fiber Q3/2 Accurel KM polypropylene (600-m i.d., 200-m wall thickness, and 0.2-m pore size) was purchased from Membrana (Wuppertal, Germany). Corning® gel-loading pipet tips (200-μl round and 0.5-mm thick) were obtained from Merck KGaA (Darmstadt, Germany).

### Instrumentation

Analyses were performed using a Waters UPLC Acquity System coupled to a Quattro Premier XE mass spectrometer (Waters Corporation, Milford, MA) with an electrospray interface (ESI) operated in positive ion mode. Chromatographic separation was achieved with an Acquity UPLC BEH C18 column (2.1 mm × 100 mm, 1.7 μm) eluted with 2 mM ammonium formate buffer with 0.1% formic acid (mobile phase A) and 0.1% formic acid in methanol (mobile phase B) at a constant flow rate of 0.3 ml/min and column oven temperature at 40°C. The following elution gradient was used: 0–0.5 min, 0% B; 0.5–7 min, 10–50% B; 7.0–7.1 min, 50–10%B; and 7.1–8.0 min, 0% B. Total chromatographic run time was 8.0 min. The injection volume was 2 μl.

The mass spectrometer was operated under multiple-reaction monitoring mode (MRM), considering three transitions for each analyte. MS settings were established as follows: desolvation gas flow rate, 1,100 l/h; cone gas flow rate, 200 l/h; desolvation temperature, 350°C; source temperature, 100°C; capillary voltage, 1,000 V. The retention times, capillary voltage, collision energy, and m/z transitions used for quantification of each analyte are indicated in Table 1.

**Table 1:** Mass spectrometry parameters for all analytes and the internal standard (quantifying transition marked with an asterisk).

**Analyte**	**Retention time (min)**	**Precusor ion, Q1 *(m/z*)**	**Product ion, Q3 (*m/z*)**	**Cone voltage (V)**	**Collision energy (V)**
DMT-*d*_6_ (IS)	2.80	195.1	63.9*	15	14
			114.9		36
			143.8		22
DMT	2.80	188.9	57.8*	25	11
			116.7		29
			143.8		17
THH	4.12	217.1	172.8	25	29
			187.9*		17
			200.0		13
HRL	5.05	215.2	130.4	50	41
			171.7*		33
			199.9		25
HRM	5.32	213.2	143.8	50	41
			169.8*		33
			198.0		25

Chromatograms were designed after extracting raw data from MassLynx™ Software (Waters Corporation, Milford, MA) and plotting them on Microsoft Excel® 2010 to improve image resolution.

### Urine Specimens

Eight authentic samples were donated from four different subjects. The first and second urine samples were collected after the individuals had participated in a typical ayahuasca ceremony. The drug-free samples were obtained from 10 non-user volunteers, pooled, and used throughout the entire method optimization and validation. After collection, samples were stored untreated at −20°C until further analysis. The study protocol was approved by the Ethics Committee of School of Pharmaceutical Sciences, University of São Paulo (ethics protocol approval number 2.267.476). Informed consent was obtained from all individual participants included in the study.

### HF-LPME Procedure

A 500-μl aliquot of urine was transferred into a 2-ml tube containing 80 mg (±10 mg) of NaCl followed by the addition of 50 μl of DMT-*d*_6_ 1 μg/ml (IS). The mixture's pH was adjusted using 200 μl of 0.1 M carbonate-bicarbonate buffer pH 10.5. After that, an 8.0-cm polypropylene hollow fiber segment previously impregnated with n-decanol and filled with 2 mM ammonium formate buffer with 0.1% formic acid (mobile phase A) using gel-loading pipet tips was introduced into the sample solution. Both ends of the fiber were sealed by pressure using pliers. The LPME system was stirred for 5 min at 2,400 rpm using a multi-vortex. After agitation, the acceptor phase was collected from the fiber lumen, transferred into a vial, and dried under nitrogen stream at 50°C. Finally, the dried residue was re-suspended with 50 μl of mobile phase A before injection (2 μl) into the UPLC-MS/MS.

### Design of Experiments

Method optimization was accomplished by design of experiments (DoE) using the tools available on the Minitab® 18 software (LLC, State College, Pennsylvania, USA).

First, a screening step was performed taking into consideration five main parameters, selected during preliminary studies (not shown) included in a fractional factorial design, resolution V (2^5−1^): (a) SLM solvent (n-octanol and n-decanol), (b) buffer pH (borate buffer pH 9.5 and carbonate-bicarbonate buffer pH 10.5), (c) buffer volume (100 and 500 μl), (d) stirring time (5 and 15 min), and (e) stirring rate (1,200 and 2,400 rpm). Sixteen random experiments were performed with a single replicate and the results were then evaluated according to the absolute response areas for all analytes.

Once these variables were screened and the significant ones were determined, a Box–Behnken design was performed with three variables at three different levels: (a) buffer pH (borate buffer pH 9.5, borate buffer pH 10.0, and carbonate-bicarbonate buffer pH 10.5), (b) buffer volume (100, 300, and 500 μl), and (c) stirring time (5, 10, and 15 min). Fifteen experiments were randomly performed with a replicate. Extraction parameters were optimized through response surface methodology (RSM) using absolute response areas.

Detailed description of experimental matrices is displayed in Tables 2, 3.

**Table 2:** Experimental matrix for the fractional factorial design.

**Experiment**	**Random order**	**SLM solvent**	**Buffer pH**	**Buffer volume (μl)**	**Stirring time (min)**	**Stirring rate (rpm)**
1	14	Octanol	10.5	100	15	2,400
2	15	Octanol	9.5	500	15	2,400
3	9	Octanol	9.5	100	5	2,400
4	8	Octanol	10.5	500	15	1,200
5	5	Octanol	9.5	100	15	1,200
6	11	Decanol	9.5	500	5	2,400
7	1	Decanol	9.5	100	5	1,200
8	4	Decanol	10.5	500	5	1,200
9	6	Decanol	10.5	100	15	1,200
10	12	Octanol	10.5	500	5	2,400
11	16	Decanol	10.5	500	15	2,400
12	3	Octanol	9.5	500	5	1,200
13	10	Decanol	10.5	100	5	2,400
14	7	Decanol	9.5	500	15	1,200
15	13	Decanol	9.5	100	15	2,400
16	2	Octanol	10.5	100	5	1,200

**Table 3:** Experimental matrix for the Box–Behnken design.

**Experiment**	**Random order**	**Buffer pH**	**Buffer volume (μl)**	**Stirring time (min)**
1	13	10.0	300	10
2	5	9.5	300	5
3	14	10.0	300	10
4	9	10.0	100	5
5	10	10.0	500	5
6	6	10.5	300	5
7	7	9.5	300	15
8	8	10.5	300	15
9	11	10.0	100	15
10	4	10.5	500	10
11	12	10.0	500	15
12	3	9.5	500	10
13	2	10.5	100	10
14	1	9.5	100	10
15	15	10.0	300	10

### Method Validation

Method validation was achieved in accordance to international recommendations (Matuszewski et al., 2003; Peters et al., 2007; United Nations Office On Drugs and Crime (UNODC), 2009). Limit of detection (LoD), limit of quantification (LoQ), selectivity, linearity, precision (intra- and inter-assay), accuracy, matrix effect (ME), recovery (RE), process efficiency (PE), and dilution integrity studies were performed. The LoD was determined as the lowest concentration with a signal-to-noise ratio (S/N) of at least 3 showing all transitions and a relative standard deviation (RSD) ≤ 20%, while the LoQ was the lowest concentration presenting a S/N ratio of at least 10 and an RSD ≤ 15%.

Specificity was evaluated in 10 urine blank samples collected from different individuals and analyzed to attest the presence or absence of any endogenous interferences. The method was also assessed for potential exogenous interfering substances through the analysis of urine samples spiked at concentrations over the calibration range of 37 common drugs and metabolites: caffeine, nicotine, cannabinoids, cocaine-related substances, amphetamines, opioids, barbiturates, antidepressants, and anxiolytics. Acceptance criteria were based on the absence of interfering peaks at the retention times of DMT, THH, HRL, HRM, and DMT-*d*_6_. IS possible interference was also assessed by analyzing a single urine sample fortified with DMT-*d*_6_ and investigated for the presence of non-deuterated DMT as an impurity.

Linearity was evaluated over a wide concentration range, from LOQ to 200 ng/ml for all analytes of interest, at five different concentrations (5, 50, 100, 150, and 200 ng/ml) with each concentration value having six replicates. The peak area ratios of the compounds to the IS were used for linear regression analysis, which was then expressed as the regression coefficients (*r*^2^ ≥ 0.99).

Precision and accuracy studies were performed by analyzing negative urine specimens spiked with the analytes at three quality control (QC) levels: low (LQC, 15 ng/ml), medium (MQC, 90 ng/ml), and high (HQC, 170 ng/ml). QCs were evaluated over 3 consecutive days, through the analysis of six replicates for each QC level. These results were expressed as %RSD, calculated with one-way ANOVA. Acceptance criteria for both intra- and inter-assay precision were RSD ≤ 20% for the LQC and ≤ 15% for both MQC and HQC. Accuracy was determined through the quantification of six replicates at each QC level and was expressed as a percentage of the known concentration, i.e., acquired mean concentration/nominal concentration × 100.

Blank samples were spiked with the analytes of interest at concentrations over the calibration range and then diluted 10- or 20-fold with 0.1 M carbonate-bicarbonate buffer pH 10.5 in order to evaluate dilution integrity. Both accuracy and precision parameters must remain within aforementioned acceptance criteria.

ME, RE, and PE experiments were assessed as described by Matuszewski et al. (2003). Three sets of experiments were prepared at LQC, MQC, and HQC with six replicates, given that each replicate was obtained from a different individual. Set 1: neat standards diluted in mobile phase; set 2: QC samples prepared by spiking blank urine samples after extraction, and set 3: represented by urine samples spiked before extraction. For RE calculation, absolute peak areas from set 3 were compared to those of set 2; for the ME, absolute peak areas from set 2 were compared to those of set 1; and for PE, absolute peak areas from set 3 were compared to those of set 1.

## Results and Discussion

### Instrumental Analysis

**Figure 1:**
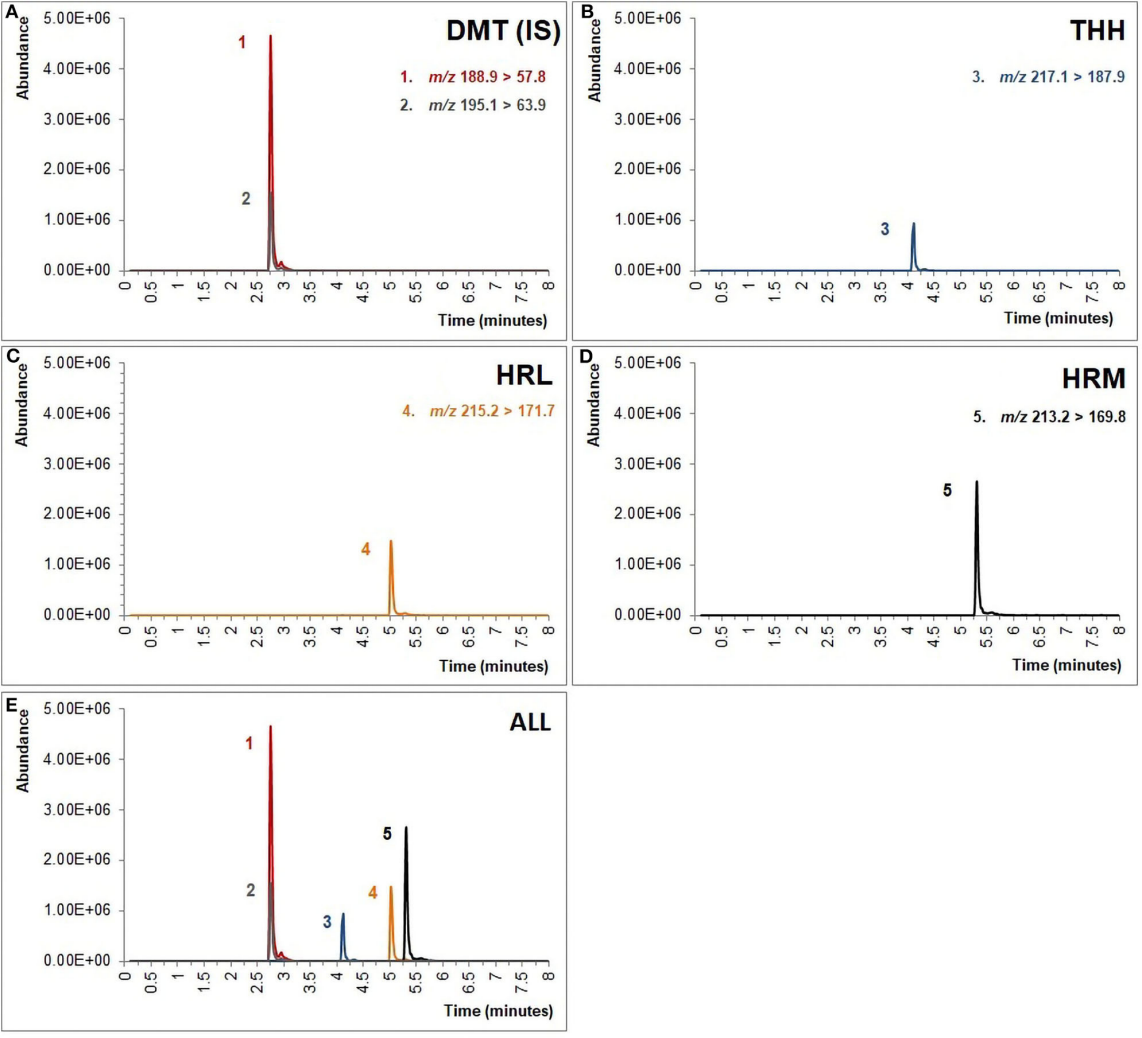
Chromatogram obtained from a fortified urine sample containing 100 ng/ml of **(A)** dimethyltryptamine (DMT) and internal standard (IS, DMT-*d*_6_), **(B)** tetrahydroharmine (THH), **(C)** harmaline (HRL), and **(D)** harmine (HRM). **(E)** All analytes are displayed together. The m/z showed above were used for quantification of the analytes.

The UPLC-MS/MS analysis, which has previously proven to be suitable for the analysis of ayahuasca tea samples (de Oliveira Silveira et al., 2020), has shown to be similarly adequate for the identification and quantification of all analytes in urine specimens using an 8-min chromatographic run as shown in Figure 1. Although chromatographic separation is not mandatory when using MRM, special attention was given to HRL and HRM separation since initial experimentation has demonstrated possible ion suppression for HRL when overlapped with HRM. Even though the focus of this study was to develop a green sample preparation method, it is clear that the greenness of this step is directly linked to the sensitivity of the subsequent chromatographic technique (Silveira et al., 2019). On the one hand, gas chromatography (GC) is considered inherently eco-friendly once organic solvents are not necessary to achieve separation as in liquid chromatography (LC) (Ghosh, 2012), but on the other hand, some strategies may be used to reduce environmental impact of LC, such as shortening the run time by reducing column length and particle size leading to an overall decrease in solvent consumption which is readily achieved with ultra-high pressure liquid chromatography (UPLC) (Kaljurand and Koel, 2012; Korany et al., 2017). Nonetheless, the replacement of organic solvents used as mobile phases in LC is also a concern when dealing with GAC requirements given that the development of greener methods often depends on improving or modifying conventional procedures (Keith et al., 2007), and for this reason, the gradient elution was modified in order to guarantee significant satisfactory method performance when using methanol as mobile phase instead of acetonitrile as the former is considered less environmentally problematic than the latter, which makes acetonitrile substitution a main challenge in GAC (De La Guardia and Armenta, 2010; Korany et al., 2017; Silveira et al., 2019).

### Design of Experiments

HF-LPME performance may be influenced by several important parameters apart from those regarding the chemical characteristics of analytes, such as the following: sample volume, pH adjustment of donor phase, volume of the pH adjustment solution (sample dilution), type of organic solvent used as SLM, agitation rate and time, acceptor solution composition, fiber segment length, and salting out (Psillakis and Kalogerakis, 2003). As urine is usually an abundant sample and of easy collection, method optimization was performed using 500 μl of the specimen, which lead to a fiber segment of 8.0 cm when using a 2.0-mL *Eppendorf* tube for optimum surface contact between phases. Considering that both LPME acceptor phase and LC mobile phase are required to have the same characteristics, i.e., a solution capable of ionizing the analytes, the acceptor solution used was composed of mobile phase A.

**Figure 2:**
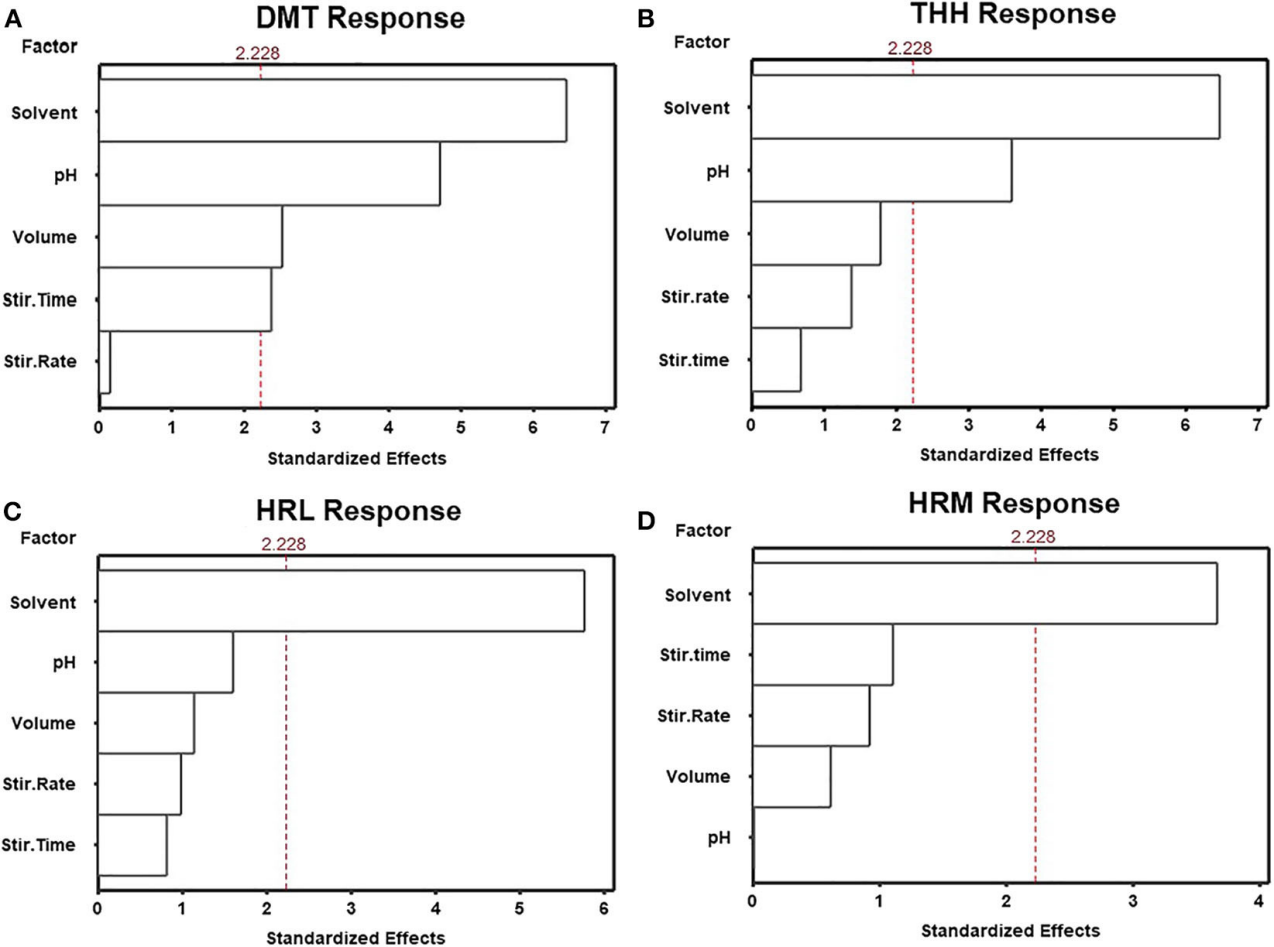
Standardized main effect Pareto charts for the fractional factorial design of **(A)** dimethyltryptamine (DMT), **(B)** tetrahydroharmine (THH), **(C)** harmaline (HRL), and **(D)** harmine (HRM).

**Figure 3:**
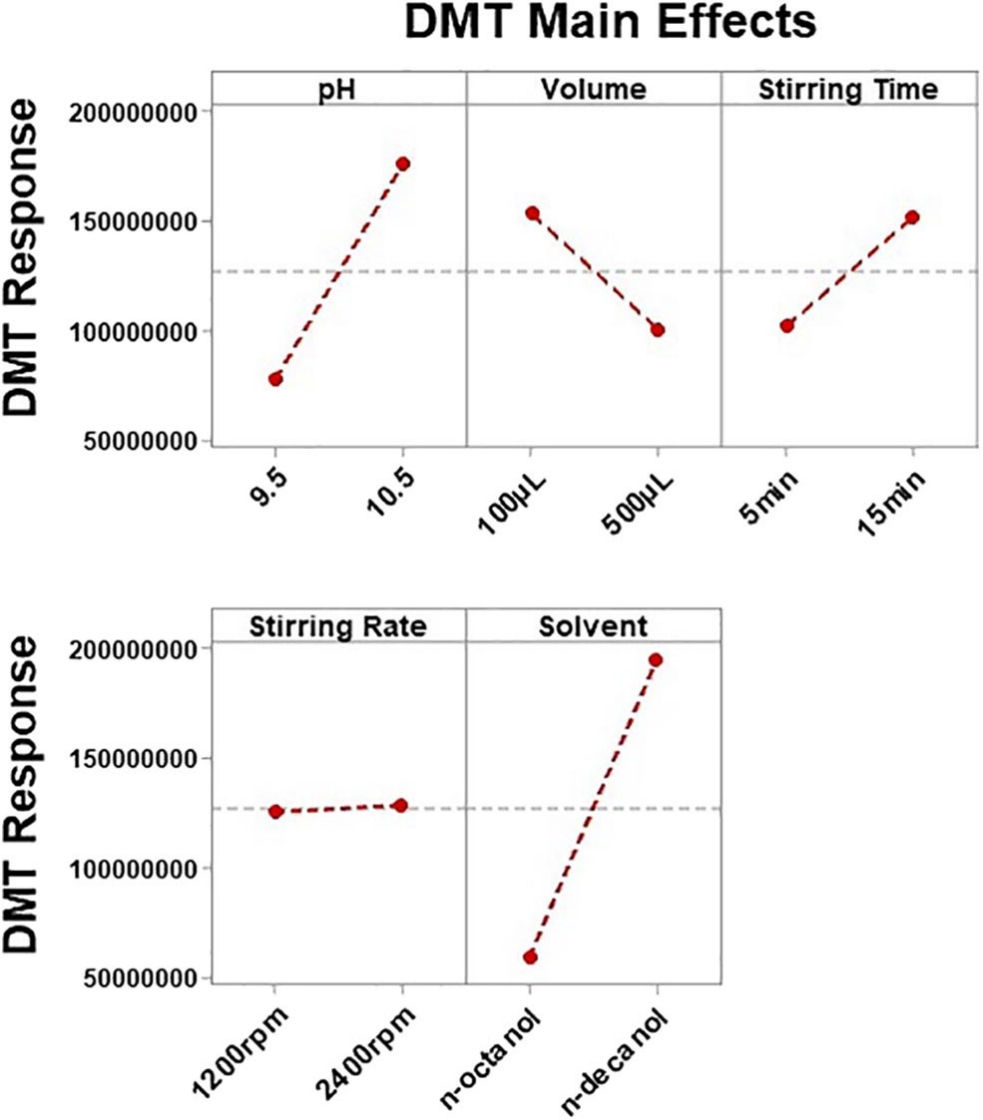
Main effect plots for dimethyltryptamine (DMT) showing the influence of each evaluated variable on absolute response areas.

Consequently, among all above-mentioned variables, five were selected during preliminary studies (data not shown) for further investigation. SLM solvent, stirring time, stirring rate, buffer pH, and buffer volume were screened using a fractional factorial design (resolution V, 2^5−1^) in order to determine variables significantly affecting method performance. Sixteen experiments were randomly performed and the results expressed as the mean of absolute response areas are displayed in Figures 2, 3. Figure 2 shows the Pareto charts for each analyte in which factors were considered of significance upon regression analysis if they revealed a *p* ≤ 0.05 (or standardized effect ≥ 2.228). According to this evaluation, only the organic solvent employed for the SLM influenced the extraction of all analytes. For this reason, the SLM composition must be carefully assessed when developing an LPME procedure since the solvent characteristics are directly associated to the extent of substance transfer through the membrane. Nonetheless, moderate polarity, viscosity, surface tensions, and boiling points are the features pursued in the search for an ideal LPME solvent (Kokosa, 2013). In this context, n-octanol and n-decanol were the solvents studied during method optimization.

THH's response was also determined by buffer pH, but only DMT's response was affected by more than three factors. Figure 3 shows how each variable influenced DMT's response. Once important parameters affecting method performance have been identified, based on the results obtained for DMT, a new optimization step was planned. Therefore, a Box–Behnken design was used to determine how these variables may be combined to promote maximum extraction response. Since n-decanol has shown to be the best solvent, not only for DMT but also for all analytes, it was incorporated in the HF-LPME procedure as well as the stirring rate of 2,400 rpm; thus, 15 Box–Behnken experiments were performed with the three remaining significant variables at three levels each (buffer pH, buffer volume, and stirring time).

**Figure 4:**
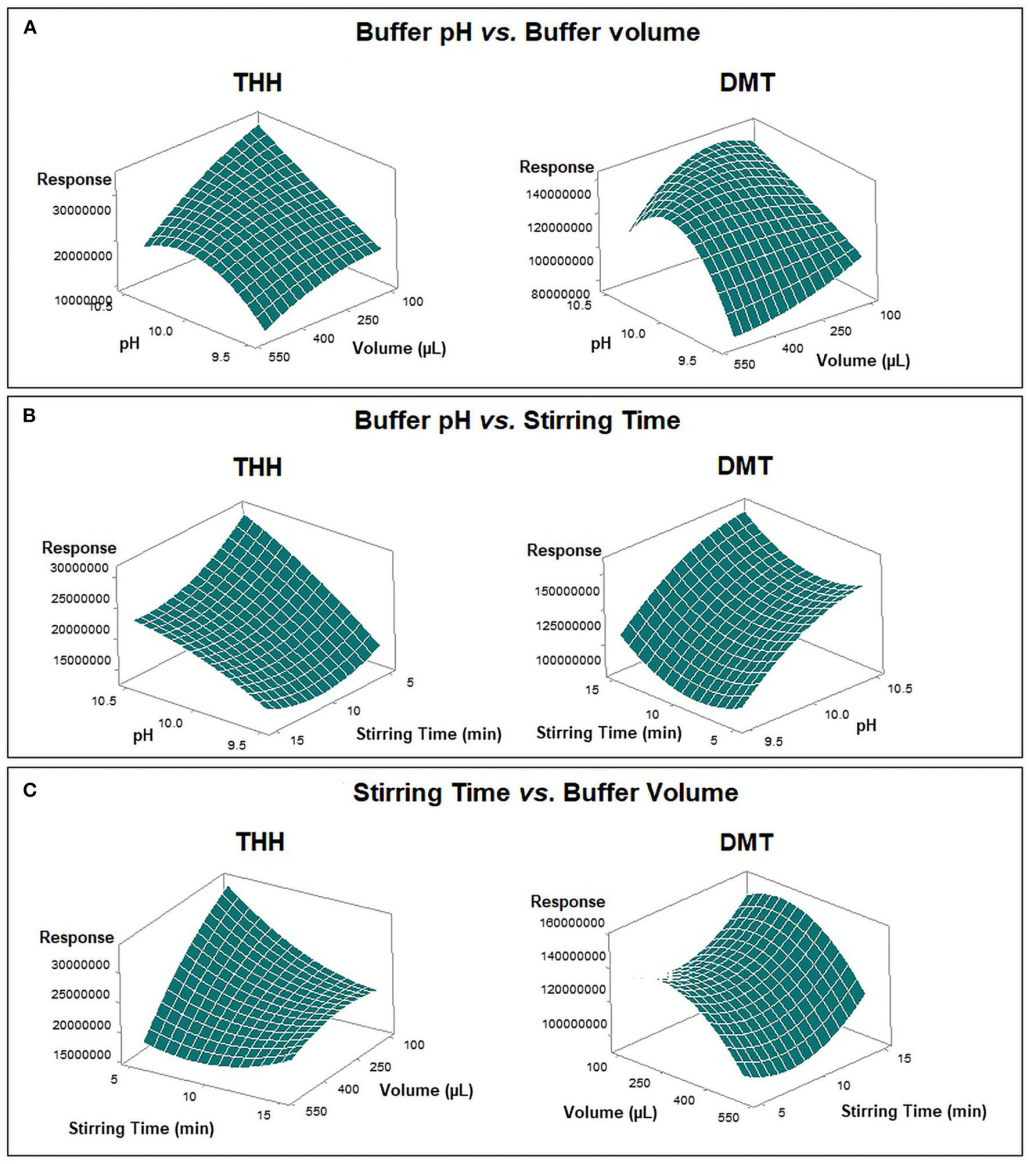
Comparative response surface plots for absolute response areas of dimethyltryptamine (DMT) and tetrahydroharmine (THH) from Box–Behnken design. **(A)** Buffer pH vs. buffer volume. **(B)** Buffer pH vs. stirring time. **(C)** Stirring time vs. buffer volume.

Despite the response by THH not being affected by buffer volume or stirring time during the first optimization step, the Box–Behnken model results were evaluated according to the best combined response for both DMT and THH. Figure 4 shows how DMT and THH responded to different factor arrangements through response surface methodology after regression analysis. The pH of the donor phase (sample) may be adjusted by using an acidic/basic solution or a buffer; the latter is preferred since it guarantees pH maintenance during the whole extraction procedure. Also, pH value plays an important role because it is responsible for keeping the analyte in its uncharged form, so the substance is able to cross the SLM. Furthermore, the volume of the pH adjustment solution is responsible for sample dilution and influences the contact surface of the sample to the fiber segment, thus promoting extraction.

**Figure 5:**
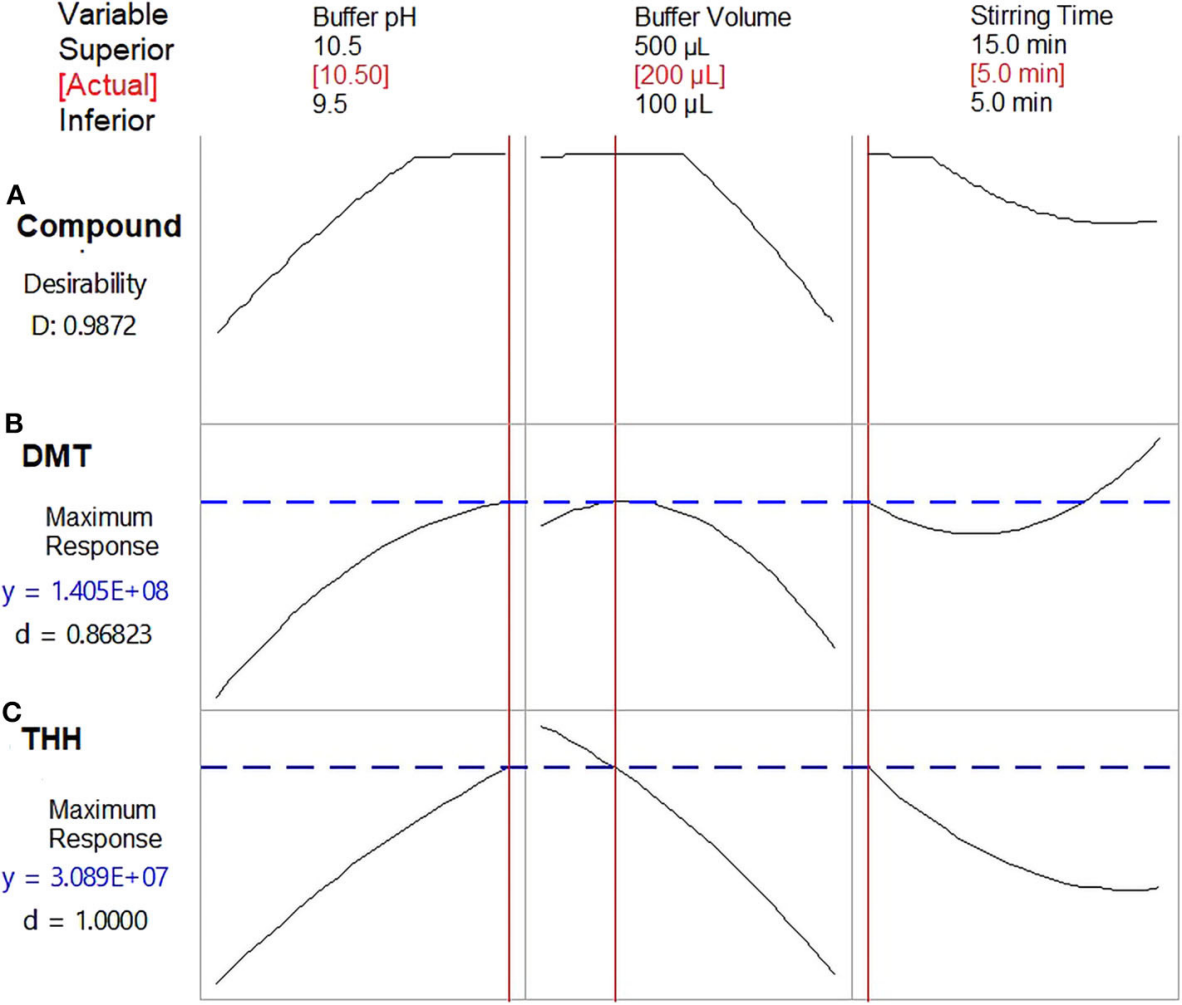
Optimization plots presenting the best combined response for dimethyltryptamine (DMT) and tetrahydroharmine (THH). **(A)** Combined variables for maximum response of both analytes; **(B)** Combined variables for maximum DMT response; **(C)** Combined variables for maximum THH response. D, desirability function. Results from Box–Behnken design.

Optimum outcomes were found for both substances when using a carbonate-bicarbonate buffer pH 10.5; however, discrepancies were observed for buffer volume (300 μl for DMT and 100 μl for THH) and stirring time (15 min for DMT and 5 min for THH). In order to obtain the maximum response with the most economic combination of these variables for both analytes, the optimization tool available on the Minitab software was applied to the acquired data; thus, a buffer volume of 200 μl and a stirring time of 5 min were set as best extraction conditions (Figure 5).

Therefore, the use of a DoE strategy led to a less time-consuming and less laborious method development when compared to univariate methodologies in which only one factor is investigated at a time while keeping the remaining unchanged (Costa et al., 2010). In this case, interactions between different parameters are not explored and the optimum arrangement may not be achieved (Costa et al., 2010). On the other hand, DoE allows for method optimization using a small number of experiments and combines the important variables in such a way that the maximum response can be obtained while minimal energy and materials are consumed, resulting in a greener extraction procedure. In our study, DoE resulted in an HF-LPME procedure using only 200 μl of a carbonate-bicarbonate buffer pH 10.5 and a fast agitation step of 5 min at 2,400 rpm, minimizing energy consumption.

### Method Validation

Liquid microextraction may be defined as a sample preparation technique employing no more than 100 μl of any organic solvent and promoting both extraction and analyte enrichment in the final residue which leads to sensitive limits of detection (Spietelun et al., 2014). In the present study, LoD (1.0 or 2.0 ng/ml) and LoQ (5.0 ng/ml) values have shown to be fit-for-purpose for all analytes. These results can be seen in Table 4 along with linearity parameters. This HF-LPME approach has proven to be linear over the range of 5.0–200 ng/ml and, since the *F*-test revealed that this method is heteroscedastic, the weighted least squares linear regression was applied resulting in a 1/*y* weighting factor for HRL and 1/*x*^2^ for DMT, THH, and HRM. All determination coefficients (*r*^2^) were above 0.99 and the relative error (RE%) of the weighted linear regression equations were never higher than 45.9% (HRL). Additionally, the least squares linear regression has resulted in negative intercept values that are not statistically different from zero, which means that the linear regression fits through the origin.

**Table 4:** Limits of detection (LoD), limits of quantification (LoQ), linear regression equation, and determination coefficients for dimethyltryptamine (DMT), harmine (HRM), harmaline (HRL), and tetrahydroharmine (THH) in fortified urine samples.

**Analyte**	**LoD (ng/ml)**	**LoQ (ng/ml)**	**Linear regression equation**	**Determination coefficient (*r*^**2**^)**
DMT	1.0	5.0	*y* = 0.0613*x* – 0.0395	0.9974
THH	2.0	5.0	*y* = 0.0121*x* – 0.0183	0.9923
HRL	2.0	5.0	*y* = 0.0347*x* – 0.0855	0.9913
HRM	2.0	5.0	*y* = 0.0848*x* – 0.1024	0.9921

The evaluation of inter- and intra-assay precision was performed during 3 consecutive days, at three QC levels with six replicates for each level. Table 5 shows that the RSD% values obtained after analysis of variance were according the acceptance criteria and ranged from 2.1% (DMT, LQC, and inter-assay) to 18% (HRL, LQC, an intra-assay). Likewise, the developed method revealed great accuracy performance ranging from 87.6% (THH and LQC) to 109.2% (THH and MQC) upon calculation using the resulting precision data.

**Table 5:** Inter-assay and intra-assay precision and accuracy for dimethyltryptamine (DMT), harmine (HRM), harmaline (HRL), and tetrahydroharmine (THH) in fortified urine samples.

**Analyte**	**QC level (ng/ml)**	**Precision (*****n*** **=** **6)**		**Accuracy (%, *n* = 6)**
		**Inter-assay (RSD%)**	**Intra-assay (RSD%)**	
DMT	15	2.1	10.2	98.5
	90	9.5	7.6	104.8
	170	8.9	7.4	103.7
THH	15	12.3	10.2	87.6
	90	9.6	10.8	109.2
	170	11.8	8.8	100.4
HRL	15	8.3	18.0	103.2
	90	11.8	11.1	99.1
	170	11.8	8.8	108.9
HRM	15	6.6	9.6	92.4
	90	5.5	11.3	95.8
	170	7.1	12.2	105.5

After ingestion, the main compounds of the ayahuasca tea are partially excreted through the urine in their unmetabolized forms (Riba et al., 2012), and their concentrations may be 20 times above the highest calibration point of the abovementioned method. For this reason, dilution integrity was validated, considering both 10- and 20-fold in two different concentrations (300 and 3,200 ng/ml). Accuracy parameters remained acceptable for all analytes (87.0–110.5%), excluding HRL which when diluted 20-fold could neither maintain its accuracy nor detect analyte in some instances. Hence, HRL quantification in authentic samples was accomplished using only a 10-fold dilution factor as it provides sufficient accuracy (86.5%).

One of the main features of the HF-LPME approach rests in its clean-up potential as the pores within the fiber wall are able to separate the uncharged analytes from the endogenous matrix interferences (Sarafraz-Yazdi and Amiri, 2010; Ghambarian et al., 2012). Regardless of the simplicity provided by dilute-and-shoot and protein precipitation (PP) methods, these alternatives are not always suitable, especially when it comes to LC-MS instrumentation. The former is prone to rather large matrix effects and instrument contamination, while the latter hardly eliminates matrix interferences other than proteins, such as phospholipids, salts, cells, small peptides, and other organic molecules present in urine specimens that can bind to detector surfaces, which may reduce the LC column lifetime and lead to low sensitivity as well as additional drawbacks generated by matrix effects (Chiu et al., 2010; Rentsch, 2016; Peters et al., 2018). Although a previous PP approach published by de Morais et al. (2018) has shown low ME, this may not be true to all LC systems with ESI interfaces, so we believe that HF-LPME may avoid these negative consequences. Additionally, PP methods previously published for the analysis of the analytes of interest in urine samples use large amounts of solvents, either acetonitrile or methyl *tert*-butyl ether (≥1.0 ml) (Zhao et al., 2012; Meyer et al., 2014; de Morais et al., 2018), to achieve sample clean-up, while the present method succeeded in accomplishing great ME and RE using volumes as low as 25 μl of an organic solvent (n-decanol). ME, RE, and PE evaluation was developed by Matuszewski et al. (2003) protocols. As suggested by Bienvenu et al. (2017), samples were selected considering a large density range. Results are can be seen in Table 6. Although most analytes have revealed a small degree of ion enhancement which may super estimate recovery values, we concluded that our method has excellent ME, RE, and PE performances.

**Table 6:** Percentage of matrix effect (ME%), recovery (RE%), and process efficiency (PE%) after submitting spiked urine samples to HF-LPME and UPLC-MS/MS analysis.

**Analyte**	**QC level (ng/ml)**	**ME (%)**	**RE (%)**	**PE (%)**
DMT	15	88.4	110.5	97.7
	90	107.1	124.8	133.7
	170	103.5	111.2	115.1
THH	15	108.7	79.2	86.1
	90	119.8	89.1	106.7
	170	112.4	97.7	109.8
HRL	15	111.7	94.8	105.8
	90	115.8	95.9	111.1
	170	112.5	99.9	112.4
HRM	15	96.0	117.9	113.2
	90	109.5	91.6	100.3
	170	101.0	102.0	103.0
DMT-*d*_6_	100	103.9	74.8	77.7

Interference studies showed no interfering peaks either by endogenous or exogenous substances at the retention times of DMT, THH, HRL, and HRM, nor was the deuterated internal standard contaminated with non-labeled DMT.

Finally, considering previously published articles on the analysis of ayahuasca alkaloids in urine specimens using LC-MS/MS, only three of them have provided detailed data regarding full validation protocols (Bjornstad et al., 2009; Mcilhenny et al., 2011; de Morais et al., 2018). The present HF-LPME method has shown higher sensitivity than most of the referred methods, except by the dilute-and-shoot alternative given by Mcilhenny et al. (2011) that achieved LoD values below 1 ng/ml. However, both LoQ figures and linearity ranges were similar in all alternatives. Simply, the screening method by Bjornstad et al. (2009) has suggested a calibration range over 200 ng/ml. Additionally, reproducibility parameters are in compliance with both the aforementioned alternatives and current validation guidelines.

### Authentic Samples

In order to prove method feasibility, eight samples were collected from four individuals. The subjects agreed in donating the specimens after having participated in a traditional ayahuasca ceremony. Three participants have ingested two different ayahuasca doses while one of them has drunk only a single dose. The first and second urine samples naturally produced by the individuals were analyzed. Information regarding the ingested amount of tea as well as the concentration of the alkaloids present in the beverage, time of consumption, and sample collection intervals were not assessed given that the alkaloid kinetics in humans is well-established, as previously published (Mcilhenny et al., 2011; Riba et al., 2012, 2015).

As displayed in Table 7, DMT concentration ranged from 67.5 to 4,120.3 ng/ml. For this last sample, a 20-fold dilution was required in order to fit the validated calibration range. THH varied from 30.6 to 908.6 ng/ml, the second most abundant alkaloid, after DMT. HRL and HRM concentrations ranged from 28.3 to 334.0 ng/ml and 30.5 to 859.4 ng/ml, respectively. HRL was the less abundant compound quantified in these urine specimens. Both the calibration range and the dilution factors validated in our study proved to be suitable for the quantification of ayahuasca alkaloids.

**Table 7:** Concentrations (ng/ml) of ayahuasca alkaloids in authentic samples after quantification by HF-LPME and UPLC-MS/MS.

**Subject**	**Sample**	**DMT**	**THH**	**HRL**	**HRM**
01	1st	1,796.8	726.3	176.6	79.6
	2nd	67.5	129.1	45.9	33.7
02	1st	820.6	70.5	41.4	149.2
	2nd	253.5	174.2	56.9	72.9
03	1st	497.6	30.6	30.5	52.7
	2nd	4,120.3	908.6	334.0	859.4
04	1st	358.0	85.3	28.3	30.5
	2nd	1,559.2	658.7	68.9	101.0

## Conclusion

Given the imminent relevance of ayahuasca researches and the consequent importance and need for the quantification of its main compounds in biological samples, the present study has described a fast and simple HF-LPME method for the determination of DMT, THH, HRL, and HRM in urine samples of ayahuasca users. After effectively applying a design-of-experiments approach, the significant factors influencing method performance were optimized to achieve the most time-, material- and energy-saving manner possible. The HF-LPME was fully validated showing excellent sensitivity, reproducibility, reduced matrix effect interferences, and outstanding recoveries. Therefore, we believe that given the fact that the sample preparation step in analytical chemistry can hardly be avoided, the eco-friendliest alternative that suits one's laboratory conditions (LC systems) must always be considered. In addition, our approach has proven to be a greener alternative when protein precipitation or dilute-and-shoot methods do not represent the most suitable option. Finally, eight authentic samples were successfully quantified by the developed method showing great applicability.

## Data Availability Statement

The raw data supporting the conclusions of this article will be made available by the authors, without undue reservation.

## Ethics Statement

The studies involving human participants were reviewed and approved by the Ethics Committee of School of Pharmaceutical Sciences, University of São Paulo (ethics protocol approval number 2.267.476). The patients/participants provided their written informed consent to participate in this study.

## Author Contributions

GS and MY contributed with the conception and design of the study. GS performed all experiments and wrote the first draft of the manuscript. FL performed the statistical analysis. VB was responsible for sample collection and consent from individuals. All authors contributed to manuscript revision, read, and approved the submitted version.

## Conflict of Interest

The authors declare that the research was conducted in the absence of any commercial or financial relationships that could be construed as a potential conflict of interest.
